# Self-assessment of Polish pharmacy staff’s readiness to promote health

**DOI:** 10.1007/s11096-020-01099-w

**Published:** 2020-08-09

**Authors:** Iwona Bojar, Beata Sarecka-Hujar, Jakub Owoc, Adrianna Pawełczak-Barszczowska, Dorota Raczkiewicz

**Affiliations:** 1 Department of Women’s Health, Institute of Rural Health, ul. Jaczewskiego 2, 20-090 Lublin, Poland; 2 Department of Basic Biomedical Science, School of Pharmacy with the Division of Laboratory Medicine in Sosnowiec, Medical University of Silesia in Katowice, Kasztanowa Str 3, 41-200 Sosnowiec, Poland; 3 Gerontology, Public Health and Education Department, National Institute of Geriatrics, Rheumatology and Rehabilitation in Warsaw, Spartańska Str 1, 02-637 Warsaw, Poland; 4 Zentiva, Warsaw, Poland; 5 Institute of Statistics and Demography, Collegium of Economic Analysis, SGH Warsaw School of Economics, Al. Niepodległości 162, 02-554 Warsaw, Poland

**Keywords:** Health promotion, Health education, Pharmacists, Pharmacy, Poland, Readiness

## Abstract

*Background* Nowadays, pharmacists are expected to focus not only on dispensing medicines but also on the wellness of the patient. In some developed countries a pharmacist is clearly defined as a health care professional that can make a contribution to improving the general health of the population. *Objective* To assess the readiness of Polish pharmacy staff to engage in health promotion and educational activities. *Setting* Community pharmacies in Poland. *Method* The study group consisted of 308 pharmacy staff (248 pharmacists and 60 pharmacy technicians) employed in Polish pharmacies. The survey questionnaire referred to three domains: systemic solutions for health promotion, readiness of pharmacy staff as a professional group to promote health, personal readiness to promote health. Responses about pharmacy staff’s readiness to promote health were scored using a 10-point scale. Scale reliability for all items (overall readiness), and for items within the three domains separately, were tested using Cronbach’s α and average inter-correlation coefficient among the items. *Main outcome measure* Pharmacy staff’s readiness to promote health (the questionnaire containing 32 items). *Results* The overall readiness of pharmacy staff to promote health was rather low (average of 4.6 ± 1.5 in 1–10 scale). The highest scores were obtained for pharmacy staff’s personal readiness to promote health (average of 5.5 ± 1.8) which was neutral on the scale. The lowest scores were obtained for systemic solutions for health promotion (average of 3.6 ± 1.4). Readiness of pharmacy staff as a professional group was ranked in the middle (average 4.8 ± 1.8). Surveyed pharmacy staff rated their readiness to promote health in the work environment significantly higher than promoting health in the local community. Female and younger pharmacy staff as well as those with job seniority of less than 5 years, or pharmacy technicians assessed their readiness to promote health significantly higher than others. Readiness to promote health was higher among pharmacy staff working in pharmacies employing up to 3 staff members and at pharmacies with over 200 customers daily. *Conclusions* The overall readiness of pharmacy staff to promote health was low, especially in the domain of systemic solutions in health promotion.

## Impacts on practice

- Polish pharmacy staff’s readiness to promote health requires further improvement especially in terms of systemic solutions and satisfying patients’ health needs, using all available methods (legal, economic, etc.).
- Financial support is needed to encourage pharmacies to engage in health promotion and health education activities, both in the workplace and the local community.
- Pharmacists should be encouraged to perceive themselves as health care professionals who can promote health and conduct health education in the local community.
- The educational curricula for pharmacists and pharmacy technicians should cover public health issues such as health promotion and health education.

## Introduction

Disease prevention and health promotion are key instruments in improving community health and are a high priority within the European Union. Nevertheless, actions aimed at fostering healthy lifestyles are complex and require the involvement of all healthcare professionals including pharmacists. Their responsibilities in this area have been dynamically evolving as pharmaceutical care directs more of its focus on the needs of a patient rather than the dispensing of drugs [1]. There is an increasing need for pharmacists to be involved in screening for health conditions, vaccination programs, and to act as primary care specialists because they are often the first point of contact for patients [2–5]. A Sudanese study demonstrated that 70% of the surveyed pharmacists were ready to engage in public health activities and expressed a desire to develop skills aimed at modifying patient behaviors [6]. In Anglo-saxon countries, pharmacists play an important role in the process of immunization and vaccination education. In the United States, pharmacists have been participating in vaccinations for influenza and measles for many years [7]. According to the American Pharmacists Association, 320,000 pharmacists have been trained to administer vaccines [8]. A study by Westrick et al. [9] indicated that most public pharmacies in the US offer at least one type of vaccine. In Poland, the competence of pharmacists do not cover vaccinations. In a meta-analysis by Isenor et al. [10] the impact of pharmacists on vaccination rates was evaluated. The study found the pharmacist’s involvement in the vaccination process as educators or vaccinators, which resulted in increased vaccine use. This may be due to the fact that the pharmacist is often the first and sometimes the only representative of the healthcare service with which the patient has contact. According to Jones et al. [11] the pharmacy profession enjoys one of the highest levels of trust among all professions. On the other hand, in a Polish study of Warsaw pharmacies, to determine when a patient seeks the help of a pharmacist, showed that 76% of respondents rarely, if ever, visited a pharmacy for purposes of a consultation with a pharmacist [12].

A pharmacist is clearly defined in some developed countries as a health care professional that can make a contribution to improving the health of the population [13–15]. British pharmacists are required to advice their clients on healthy lifestyle, well-being and provide information on how to stop smoking [13]. In Scotland, community pharmacists advise on sexual health [14]. In 2017, Public Health England, a governmental agency, published a document outlining a wide variety of actions that play a vital role in protecting and improving population health that should be performed by pharmacists [15].

The Pharmaceutical Group of the European Union describes the role of a pharmacists in improving public health and indicates ways to achieve such goal to benefit individuals, families and communities [16]. According to the Polish Act “Pharmaceutical Law”, pharmacies are defined as institutions of public health protection [17]. There is however, a need for legal and systemic changes in the healthcare system that would allow pharmacy personnel the full usage of their potential in delivering pharmaceutical services related to public health such as promoting healthy behaviors or primary and secondary disease prevention. The literature recommends involving pharmacists in a wide range of healthcare services: prevention, health education or support in overcoming addictions [18–21]. It is also suggested that they may contribute to reducing health inequalities [22], reducing costs and increasing the effectiveness of treatments and improving medical care in general [5]. Pharmacists from various countries are aware of the need to engage in health promotional activities, but also of the need for continuous education in that respect [23]. This is especially the case with community pharmacist who participate in such training less frequently than the hospital ones [24].

From 1990 to 2012, the number of pharmacies in Poland increased over 3.5 times. During this period, chain pharmacies began to develop rapidly, forcing small family pharmacies out of the market. Since 2012, a decrease in the number of pharmacies has been noted, especially from 2017, when new laws were adopted. In order for a pharmacy to be opened there must be at least 3000 local residents and must be at least 500 m from an existing one, and its owner must be a pharmacist or a company of pharmacists [25]. According to Statistics Poland, there are over 2600 patients per pharmacy in Poland, which is over 1.6 times less than the European average [26]. In Poland, there are 70 pharmacists per 100,000 residents, while this average is 82 for OECD countries. Thus, there are too many pharmacies in Poland and too few pharmacists (on average 1.8 pharmacists per one pharmacy, while the European average is 2.4) [26]. The duration of pharmaceutical studies in Poland is 5.5 years with six months of professional practice in a pharmacy. Pharmacy staff also include pharmacy technicians who have completed a 2.5-year course with another 2 years of practice in a pharmacy, and can perform similar activities as pharmacists with the exception of dispensing narcotic and psychotropic substances, but they must work under the supervision of a pharmacist.

### Aim of the study

The objective of the present study was to evaluate readiness of pharmacy staff working in Poland to perform health promotion and education activities.

### Ethics approval

The present survey, involving human participants, was in accordance with the ethical standards of the institutional bioethics committee and the Helsinki declaration (1964). In Poland, surveys are not considered as medical research and do not require the formal approval of a Local Ethics Committee. Participation in the study was anonymous and voluntary, all pharmacy staff gave their verbal consent.

## Methods

### Study group

The study group consisted of pharmacy staff—pharmacists and pharmacy technicians—who have a direct contact with patients and dispense medications in Poland. The survey was conducted in 2017 in community pharmacies in the Lublin region in Poland. Community pharmacies in this region are representative of community pharmacies in the other 15 regions in Poland, as they all function in the same manner and are governed by the same legal regulations. In 2017, in the Lublin region there were 865 pharmacies listed on the National Health Fund (NHF) Lublin Region website. Due to some financial and organizational limitations, 140 pharmacies from this list were selected for the study, which constitutes 16% of all pharmacies in Lublin region. A systematic sample of pharmacies was selected, where every sixth pharmacy from the NHF list was chosen. Each selected pharmacy was sent 5 copies of the survey questionnaire and asked to fill it in by the pharmacy staff. The total of 368 survey questionnaires were sent back to us. The response rate was 52%. A total number of 308 correctly completed questionnaires were included in the study.

### Survey questionnaire

The survey questionnaire contained 3 sections. The first section referred to the characteristics of pharmacy staff: gender, age, level of education, job seniority. The second section referred to the characteristics of pharmacies: number of staff, location, number of customers daily. In the third section, we adopted the Scale of Social Readiness by Gaś [27] to self-assess Polish pharmacy staff’s readiness to promote health. Generally, this scale is used to research various social groups’ attitudes to the prevention of different social issues. Our questionnaire included 32 items in three domains: systemic solutions for health promotion (16 items), readiness of a professional group (10 items) and personal readiness to promote health (6 items). Systemic solutions are related to relevant legal acts, certain activities undertaken by pharmacists, organizations and institutions, cooperation between various professional groups and financial support of health promotion in pharmacies in Poland. The readiness to promote health by the pharmacy staff as a professional group is determined by the following questions: are pharmacists as a professional group prepared to promote health in terms of knowledge and methodology, and whether they have motivation, space, organizational and interpersonal skills. In turn, personal readiness of pharmacy staff to promote health is demonstrated by the following questions: are they personally ready and committed, and whether they have organizational and interpersonal skills to promote health.

All items were rated on a 1−10 scale where: 1 equaled a ‘definitely no’ and 10 equaled a ‘definitely yes’. The middle of the scale was set at 5.5 so replies statistically significantly lower than 5.5 were considered as negative, replies statistically significantly higher than 5.5 were considered as positive and replies statistically not significantly different from 5.5 were considered as neutral (neither positive nor negative).

We tested scale reliability for all 32 items and for items within the three domains separately: systemic solutions for health promotion, readiness of a professional group and personal readiness to promote health. We used Cronbach’s α and average inter-correlation coefficient among the items r.

The total scale of pharmacy staff’s readiness to promote health had α = 0.956 and r = 0.426. The individual domains had the following scores: systemic solutions for health promotion at α = 0.938 and r = 0.503, readiness of the professional group to promote health at α = 0.928 and r = 0.585 and personal readiness to promote health at α = 0.899 and r = 0.629. The factor analysis confirmed the extraction of three domains that explained 61.7% of the total item variance. Cronbach’s α values above 0.7, strong inter-correlations among the items and high percentage of total item variance indicate high reliability and internal coherence of the total scale and the three domains.

### Statistical methods

The statistical analysis was conducted with STATISTICA 12 software (Statsoft, Poland). The absolute numbers (n) and percentages (%) were estimated for the categorical variables. Arithmetic mean (M) and standard deviation (SD) were estimated for continuous variables. The following statistical tests were used:

- one sample *t* test against a value of 5.5 to check whether readiness to promote health is neutral or statistically significantly positive or significantly negative,
- two paired samples *t* test to compare readiness to promote health between at workplace and in local community,
- two unpaired samples *t* test to compare readiness to promote health between men and women,
- F-test analysis of variance to compare readiness to promote health between 3 levels of education, 4 intervals of job seniority, 3 intervals of number of employees, 4 locations of pharmacies, 3 intervals of average number of customers daily,
- Pearson’s correlation coefficient r to correlate readiness to promote health with age of pharmacy staff.

The significance level was assumed at 0.05.

## Results

### Characteristics of pharmacy staff and pharmacies

The characteristics of pharmacy staff and pharmacies are presented in Table 1. The surveyed group consisted of pharmacy technicians (19.5%), masters of pharmacy (63%), postgraduate education, professional specialization or Ph.D. in pharmacy (17.5%).

**Table 1:** Characteristics of pharmacy staff and pharmacies studied

Characteristics of	Variable, parameter	Category/IU	Result
Pharmacy staff	Total sample, N	−	308 (100.00)
	Gender, n (%)	Women	279 (90.58)
		Men	29 (9.42)
	Age, M ± SD	Years	38.0 ± 11.1
	Level of education, n (%)	Pharmacy technicians	60 (19.48)
		Masters of pharmacy	194 (62.99)
		Postgraduate education, professional specialization or Ph.D.	54 (17.53)
	Job seniority, n (%)	Below 5 years	72 (23.38)
		5−14 years	136 (44.16)
		15−25 years	48 (15.58)
		Over 25 years	52 (16.89)
Pharmacies	Number of staff, n (%)	Up to 3	50 (16.23)
		4−5	141 (45.78)
		Over 5	117 (37.99)
	Location, n (%)	Near a healthcare provider	182 (59.09)
		Urban area with high pedestrians traffic	61 (19.81)
		Residential area	29 (9.42)
		Other	36 (11.69)
	Daily average number of clients, n (%)	Below 100	97 (31.49)
		100−200	153 (49.68)
		Over 200	58 (18.83)

The pharmacy staff’s age ranged from 21 to 65 with the average of 38.0 ± 11.1 years. The participants were mostly women (91%), with a university degree in pharmacy (63%), with 5–14 years of job seniority (44%). Most pharmacies employed 4–5 employees (46%) and were located near a healthcare provider (59%). Half of all pharmacies served 100–200 customers daily on average.

### Assessment of pharmacy staff’s readiness to promote health

The replies to questions that referred to systemic solutions for health promotion had an average of 2.7–4.4 points in 1–10 scale, which was statistically significantly below the middle i.e. were rated negatively (Table 2). The lowest ratings referred to: financial support for health promotion, pharmacy leaders in health promotion, cooperation between professional groups in health promotion and using research results in health promotion (average scores of 2.7–3.1). Participants rated higher: ideas, examples and technical solutions for health promotion (average scores of 3.4–3.5). The highest ratings referred to: strategy, legal regulations, concept of health promotion, as well as institutional and professional support (average scores of 4.0–4.6).

**Table 2:** Assessment of systemic solutions for health promotion

Question	M	SD	t^a^	*p*
Is implementation of executive regulations and other guidelines for pharmacy staff in health promotion enforced by the pharmacy’s supervision body or professional associations?	4.6	2.6	− 6.075	< 0.001
Is there a clearly defined (in your professional environment of pharmacy staff) concept of health promotion that includes prophylactics, local health policy and health education?	4.4	2.1	− 9.193	< 0.001
Are there institutions or organizations that provide professional training for pharmacy staff in the area of health promotion, including health education?	4.3	2.2	− 9.573	< 0.001
Are there clear regulations that define and support a role of pharmacy staff in health promotion?	4.2	2.1	− 10.864	< 0.001
Does pharmacy professional association take actions aimed at improving competence of pharmacy staff as health promoters and at setting legal-organizational framework for such activities?	4.1	2.2	− 11.168	< 0.001
Is there any coordinated work among pharmacy staff on strategy for health promotion by pharmacy staff on a regional, local and institutional level?	3.9	2.3	− 12.209	< 0.001
Are there any institutions/organization that try in an orderly manner to cooperate with pharmacy staff and support them in activities aimed at health promotion?	3.8	2.1	− 14.207	< 0.001
Do pharmacy staff have benchmark solutions for activities aimed at health promotion that can be used to work out their own health-educational programs?	3.5	2.0	− 17.550	< 0.001
Do pharmacy staff have a working concept of coordinated activities aimed at assessing quality and effectiveness of health promotion activities performed by them?	3.4	1.9	− 19.397	< 0.001
Is there an effective system of support for pharmacy staff that delivers technical concepts for health promotion, technical support and information materials?	3.4	1.8	− 20.475	< 0.001
Is it a common practice among pharmacy staff to use results of epidemiological and demographic research to plan activities in the area of health promotion and information?	3.1	1.6	− 26.325	< 0.001
Are there among pharmacy staff on a regional and local level people who may be considered spokespersons or leaders of the “Pharmacy promoting health” concept?	3.1	1.8	− 23.400	< 0.001
Does pharmacy professional association take any action aimed at working out a financial framework for such activities?	3.1	1.8	− 23.400	< 0.001
Is there cooperation between local communities, local and central administration, and pharmacy staff focused on prophylactics/prevention of drug dependence and addiction, and on monitoring self-treatment?	3.0	1.8	− 24.375	< 0.001
Is there a system of financial support for pharmacies in performing health promotion activities, including health education, at workplace?	2.7	1.9	− 25.863	< 0.001
Is there a system of financial support for pharmacies in performing health promotion activities, including health education, with local communities?	2.7	1.9	− 25.863	< 0.001

Scale 1–10 where: 1 equaled to ‘definitely no’ and 10 equaled to ‘definitely yes’

*M *mean, *SD *standard deviation

^a^One sample *t* test against a test value of 5.5

Table 3 shows readiness of pharmacy staff as a professional group to promote health. The highest ratings referred to positive motivation to actively promote health including health education both at workplace and in a local community (average score of 5.7 and 5.5, respectively, not statistically significantly different from the middle of scale). Lower ratings referred to ‘knowledge and its communication’ and ‘conditions (premises, organizational and interpersonal, professional group’s initiatives to promote health), that were rated significantly below the middle of scale.

**Table 3:** Assessment of readiness to promote health by pharmacy staff as a professional group

Question	At workplace				In local community				Workplace versus local community	
	M	SD	t^a^	*p*	M	SD	t^a^	*p*	t^b^	*p*
Do pharmacy staff show positive motivation for active involvement in health promotion and health education?	5.7	2.5	1.404	0.160	5.5	2.3	0.001	0.999	2.197	.029
Are pharmacy staff ready to promote health and health education in terms of their knowledge?	5.1	2.3	− 3.052	.002	4.9	2.4	− 4.387	< 0.001	2.359	.019
Do pharmacy staff initiate activities aimed at increasing their readiness to promote health?	5.1	2.1	− 3.343	.001	4.9	2.4	− 4.387	< 0.001	2.186	.030
Are pharmacy staff ready to promote health and health education in terms of methodology *(objectives, forms, methods, influencing, methods of diagnosing patient/client, methods of evaluation and other)*?	4.8	2.2	− 5.584	< 0.001	4.6	2.2	− 7.180	< 0.001	3.770	< 0.001
Do pharmacy staff have appropriate conditions (premises, organizational, interpersonal) to promote health and health education?	4.1	2.2	− 11.168	< 0.001	3.4	1.9	− 19.397	< 0.001	7.455	< 0.001

Scale 1−10 where: 1 equaled to ‘definitely no’ and 10 equaled to ‘definitely yes’

*M *mean, *SD *standard deviation

^a^One sample *t* test against a test value of 5.5

^b^Two paired samples *t* test

We found that the pharmacy staff preferred promoting health at the workplace rather than in the local community (p < 0.05).

The questions that referred to self-perceived readiness to promote health are presented in Table 4. The pharmacy staff rated highest their readiness to recognize and meet health expectations of customers, social need for health promotion and interpersonal relations facilitating such services (average scores of 5.8-6.0). They rated lower their organizational abilities and proper facilities (4.9) and readiness to perform such tasks with local community (5.1).

**Table 4:** Assessment of personal readiness to promote health

Question	M	SD	t^a^	*p*
Do you think you are ready to recognize and meet health expectations of patients/pharmacy clients to a larger extent than it is necessary for regular buy/sell relations?	6.0	2.2	3.989	< 0.001
Do social attitudes and personal relationships at workplace favor health promotion and health education?	5.9	2.4	2.925	.003
Do you think you are effective at health promotion?	5.8	2.0	2.632	.008
Is the level of your readiness to promote health at your workplace sufficient for effective actions?	5.4	2.0	− 0.877	0.380
Is the level of your readiness to promote health at your local community sufficient for effective actions?	5.1	2.1	-3.343	.001
Do you have appropriate premises and organizational setting for health promotion activities?	4.9	2.4	-4.387	< 0.001

Scale 1–10 where: 1 equaled to ‘definitely no’ and 10 equaled to ‘definitely yes’

*M *mean, *SD *standard deviation

^a^One sample *t* test against a test value of 5.5

In Table 5 we calculated the overall ratings of the three domains: the systemic solutions for health promotion (as a mean of 16 items in this domain), the readiness of a professional group (as a mean of 10 items in this domain) and the personal readiness to promote health (as a mean of 6 items in this domain). We also calculated an overall score of the assessment of pharmacy staff’s readiness to promote health as a mean of 3 domains mentioned above.

**Table 5:** Assessment of pharmacy staff’s readiness to promote heath versus pharmacy staff’s and pharmacies’ characteristics

Characteristics of	Variable	Category, parameter	Systemic solutions	Readiness of professional group	Personal readiness	Overall score
Pharmacy staff	Total sample	M ± SD	3.6 ± 1.4	4.8 ± 1.8	5.5 ± 1.8	4.6 ± 1.5
	Gender	Women, M ± SD	3.7 ± 1.4	4.9 ± 1.8	5.5 ± 1.8	4.7 ± 1.5
		Men, M ± SD	2.7 ± 1.0	4.5 ± 1.4	5.7 ± 1.5	4.3 ± 1.1
		p (t)	< 0.001	0.247	0.555	0.168
	Age (years)	r	− 0.163	− 0.145	− 0.222	− 0.199
		*p*	.004	.011	< 0.001	< 0.001
	Level of education	Pharmacy technicians, M ± SD	4.4 ± 1.6	5.5 ± 1.8	5.9 ± 2.0	5.3 ± 1.6
		Masters of pharmacy, M ± SD	3.4 ± 1.4	4.7 ± 1.9	5.3 ± 1.8	4.5 ± 1.5
		Postgraduate education, professional specialization or Ph.D., M ± SD	3.4 ± 1.2	4.7 ± 1.5	5.9 ± 1.6	4.7 ± 1.2
		*p* (F)	< 0.001	.008	.021	.002
	Job seniority (years)	Below 5, M ± SD	4.5 ± 1.4	5.9 ± 1.5	6.4 ± 1.6	5.6 ± 1.3
		5–14, M ± SD	3.1 ± 1.2	4.3 ± 1.8	5.2 ± 2.0	4.2 ± 1.5
		15–25, M ± SD	3.7 ± 1.5	4.6 ± 1.4	5.7 ± 1.2	4.7 ± 1.2
		> 25, M ± SD	3.5 ± 1.3	5.0 ± 1.8	5.0 ± 1.8	4.5 ± 1.4
		*p* (F)	< 0.001	< 0.001	< 0.001	< 0.001
Pharmacies	Number of staff	Up to 3, M ± SD	4.1 ± 1.5	6.3 ± 1.4	7.1 ± 1.4	5.8 ± 1.1
		4–5, M ± SD	3.3 ± 1.3	4.4 ± 1.8	5.3 ± 1.7	4.4 ± 1.5
		> 5, M ± SD	3.7 ± 1.5	4.7 ± 1.7	5.1 ± 1.7	4.5 ± 1.4
		*p* (F)	.002	< 0.001	< 0.001	< 0.001
	Location	Near a healthcare provider, M ± SD	3.6 ± 1.2	4.8 ± 1.7	5.5 ± 1.8	4.6 ± 1.4
		Urban area with high pedestrian traffic, M ± SD	3.7 ± 1.7	4.7 ± 2.0	5.5 ± 1.9	4.6 ± 1.7
		Residential area, M ± SD	3.3 ± 1.6	5.1 ± 1.8	5.8 ± 2.1	4.7 ± 1.6
		Other, M ± SD	3.5 ± 1.7	4.9 ± 2.2	5.6 ± 1.8	4.7 ± 1.7
		*p* (F)	0.497	0.718	0.864	0.995
	Daily average number of clients in a pharmacy	Below 100, M ± SD	3.2 ± 1.2	4.2 ± 1.8	4.9 ± 2.0	4.1 ± 1.5
		100–200, M ± SD	3.8 ± 1.5	5.1 ± 1.8	5.9 ± 1.7	4.9 ± 1.5
		> 200, M ± SD	3.8 ± 1.5	5.1 ± 1.5	5.7 ± 1.3	4.9 ± 1.3
		*p* (F)	.003	< 0.001	< 0.001	< 0.001

Scale 1–10 where: 1 equaled to ‘definitely no’ and 10 equaled to ‘definitely yes’

*M* mean, *SD* standard deviation, *r* Pearson’s correlation coefficient, *t* Student’s *t* test for means in two unpaired samples, *F* analysis of variance *F* test for means in more than two unpaired samples

The overall readiness of pharmacy staff to promote health was low (average of 4.6 ± 1.5, t = −14.044, *p* < 0.001). The highest scores referred to pharmacy staff’s personal readiness to promote health (average of 5.5 ± 1.8, t = 0.271, *p* = 0.786) which was in the neutral area on the scale. The lowest scores referred to systemic solutions for health promotion (average of 3.6 ± 1.4, t = −23.227, *p* < 0.001). Readiness of professional group to promote health was ranked in the middle (average of 4.8 ± 1.8, t = − 6.521, *p* < 0.001).

There were statistically significantly positive correlations: between ratings of systemic solutions for health promotion and readiness of pharmacy staff as a professional group (r = 0.695, *p* < 0.001), between ratings of systemic solutions for health promotion and personal readiness (r = 0.542, *p* < 0.001), between personal and professional group’s readiness (r = 0.800, *p* < 0.001). It shows that higher scores in one domain translated into higher average scores in other domains of readiness to promote health.

### **Correlation of pharmacy staff and pharmacies characteristics with/to pharmacy staff’s readiness to promote health**

In Table 5 we used the overall scores for the whole questionnaire and scores of 3 domains to correlate them with the respondents’ and pharmacies’ characteristics. Women rated the systemic solutions for health promotion higher than men (3.7 vs. 2.7, p < 0.001), however the assessment of other domains did not correlate to gender (*p* > 0.05). Age of respondents correlated negatively with their overall readiness to promote health and assessment of the three domains: systemic solutions, personal readiness and readiness of a professional group (r < 00, *p* < 0.05). The younger the pharmacists the higher were the average scores of readiness to promote health.

Pharmacy technicians rated significantly better than pharmacists with university or post-university education the following: systemic solutions (4.4 vs. 3.4 or 3.4, *p* < 0.001), readiness of the professional group (5.5 vs. 4.7 or 4.7, *p* = 0.008) and overall readiness (5.3 vs. 4.5 or 4.7, *p* = 0.021). Respondents with the shortest job seniority (up to 5 years) rated overall readiness to promote health and in three domains, significantly better than others (*p* < 0.001).

Pharmacy staff from small pharmacies (up to 3 employees) rated overall readiness to promote health and in three domains, statistically significantly better than pharmacy staff from larger pharmacies (4–5 employees or > 5 employees). No correlation was found between location of a pharmacy and: overall readiness, systemic solutions, personal readiness and readiness of the professional group (*p* > 0.05). Pharmacy staff from pharmacies serving below 100 clients a day rated overall readiness to promote health and in three domains, statistically significantly lower than pharmacy staff from pharmacies serving 100–200 or more than 200 clients a day.

## Discussion

The data on how pharmacists perceive their role in health promotion, education and disease prevention is necessary for developing an effective public health program for pharmacies. Such a discussion intensified in the late 1990s. A study by O’Loughlin et al. [28] from Canada indicated that very few pharmacists discussed disease-prevention with their customers on a routine basis. Nevertheless, over 90% of them believed that adding preventive healthcare to their practices would be very important but was limited by lack of time and skills.

In the present study we aimed to determine the readiness of Polish pharmacists to promote health and provide health education.

The areas that participants were least positive about were as follows: financial support, leadership, cooperation between professions and failure to apply the results of scientific research. The involvement of pharmacists in health promotion and preventive measures may support public health by lowering costs of patient care and therapies. Poland strives for pharmacists to extend their role in public health in areas such as promotion of healthy behaviors as well as primary and secondary prevention of diseases. Involvement of pharmacists also helps improve patient compliance and the lowering of risks and drug related adverse events and interactions. Positive motivation towards active involvement in health promotion (at workplace and within local communities) was highly rated by the pharmacists in the study. Other aspects of their readiness such as professional and methodological knowledge, appropriate premises or soft skills were rated significantly lower. The readiness to promote health at workplace is significantly higher than within a local community.

The study by Mohamed et al. [6] indicated that almost 90% of pharmacists provided patients with information on healthy diet while 80% educated them on obesity and body mass reduction. In Kuwait, pharmacists are likely to involve in advising patients on recommended medication use, potential side effects and lifestyle, although less so to advice on healthy behaviors [29]. However, while a vast majority is willing to learn more about promoting health, they indicate lack of time as a major obstacle. Pharmacists from Riyadh in Saudi Arabia are one of the most trusted professions in understanding needs of society [30]. This is achieved through frequent interactions with patients and customers because pharmacists are often the first and sometimes the only health care professional for many. Nevertheless, they are reluctant to educate patients on oral health although they have proper knowledge [30].

The pharmacy staff in the present study assigned the highest scores to their own “readiness to promote health” followed by “social attitude/interpersonal relationships at workplace that favor health promotion”. The lowest scores were assigned to appropriate premises and conditions to promote health and pharmacy staff’s own readiness to promote health outside of workplace.

The lowest ratings were assigned to “appropriate premises and organizational conditions for health promotion” and “readiness to promote health outside of workplace”. Review of literature indicates that pharmacists play an important role in promoting health, especially when it comes to cardiovascular diseases (CVD), despite some barriers in pharmacist-patient communication [31]. Other obstacles related to CVD prevention and health education by pharmacists included a lack of educational materials [32]. The important factor in advising patients is adequate time. It can be neither too short because a patient may think the case is trivialized, nor too long because a patient may feel bored. On the other hand, a higher number of customers per pharmacist, or a desire to fill a prescription quickly may hamper proper counselling [33]. Insufficient time devoted by pharmacists was indicated as the main reason for patients’ dissatisfaction with advice received from pharmacists in Korea [34]. Nevertheless, in the group of 252 patients and 620 pharmacists, 34% and 50%, respectively, said they were satisfied with counselling. A Canadian study that investigated actual level of pharmacists’ involvement in health promotion and disease prevention found that the major barriers included lack of: time, coordination with other professionals, staff and resources, and financial compensation [35].

In our study female pharmacy staff rated systemic solutions for health promotion statistically significantly better than male pharmacy staff. In Poland, most pharmacists are women (almost 83% pharmacists and 95% pharmacy technicians according to Zappa Report [26]), as in our sample. Another finding was that the age of pharmacy staff surveyed was associated with some of the ratings—the younger the persons the better they rated readiness to promote health. This may be due to the fact that youths are more socially aware, modern educated, with better access to information, full of enthusiasm and less routine. Systemic solutions, readiness of pharmacy staff as a professional group and the overall readiness were rated higher by pharmacy staff with a secondary level of education. There were no correlations between pharmacy location and overall readiness, systemic solutions, readiness of pharmacy staff as a professional group and personal readiness to promote health.

The increasing role of pharmacists led to develop a new term—*Pharmaceutical Public Health* [36]. It is defined as pharmacists’ input into social health by application of their knowledge and skills to prevent diseases, prolong life, promote, protect and improve health. The *Pharmaceutical Public Health* services should be as important as pharmaceutical care in the healthcare systems. However, it is crucial to properly identify health needs of people in order to develop the concept of *Pharmaceutical Public Health.*

There is a need in Poland to implement a modern system of pharmaceutical services that would take into account a unique role of a pharmacy as a point of delivering health services. Such implementation requires, proper legal regulations complemented with organizational and financial mechanisms. The results of our research indicate the need for pharmacy staff’s education in the field of health promotion and health education. In our opinion, this education should already begin at university. The study by Rodis et al. [37] demonstrated that students from pharmacy schools in the USA, who were educated in the field of health promotion, positively evaluated this education program. Moreover, pharmacy students from Gdańsk (Poland) were found to correctly understand the concept of “public health” [38]. The students’ knowledge of this topic, according to their self-assessment, was at an average level. Interestingly, the students assessed the involvement of pharmacists in the protection of public health as insufficient [38]. Currently, a great commitment from pharmacy students throughout Poland in health-promoting campaigns can be observed. During such events, students demonstrate how to measure blood pressure or blood glucose correctly. The importance of health promotion and disease prevention is especially visible during an epidemic status. The prevention of e.g. a virus spreading minimizes the number of cases and, in turn, does not cripple the health service.

The strength of our study is a sizeable study sample in which we evaluated the systemic solutions, readiness of pharmacy staff as a professional group, self-assessment of pharmacy staff’s personal readiness to promote health and to perform health education.

Our study was performed within one region of Poland. However, our sample is representative in terms of the manner/conditions of functioning and legal acts as well as gender of pharmacy staff.

A lack of opportunity for open responses on how pharmacy staff really see their role in health promotion, whether they want to perform such activities and what they believe are the biggest barriers for ideal health education may be one of the study’s limitation. We also did not ask surveyed pharmacists about the role they play in the pharmacy; it is not therefore clear how many of them were owners of pharmacies or pharmacy managers. Another limitation of our study is a lack of assessment of the demands and expectations of various groups of patients/customers of pharmacies regarding health promotion and education by pharmacy staff. However, it will be a perfect complement to our future research.

## Conclusions

General readiness of pharmacy staff to promote health was low-significantly below the scale midpoint. The lowest ratings referred to systemic solutions for health promotion while the highest to personal readiness to promote health. Readiness of pharmacy staff as a professional group ranked in the middle. Pharmacy personnel surveyed in the study rated the readiness to promote health in work a environment to a greater extent than in the local community. Women, younger respondents, respondents with job seniority below 5 years and respondents with secondary education assessed their readiness to promote health better than others. Readiness to promote health was higher among pharmacy staff working in pharmacies employing up to 3 staff members and at pharmacies with over 200 customers daily.
